# Public Awareness About Sexually Transmitted Diseases in Taif, Saudi Arabia

**DOI:** 10.7759/cureus.42302

**Published:** 2023-07-22

**Authors:** Ibrahim A Aseeri, Mansour N AlOtaibi, Waleed J Alzahrani, Mohammed A Althomali, Hattan A Alattar, Ahmed F Althobity

**Affiliations:** 1 Faculty of Medicine, Taif University - College of Medicine, Taif, SAU; 2 Department of Surgery, Taif University - College of Medicine, Taif, SAU

**Keywords:** preventive medicine, gonorrhea and chlamydia, viral hepatitis b, hpv infection, hiv transmission, public health awareness, sexual transmitted diseases

## Abstract

Introduction: Islamic culture does not tolerate homosexuality and extramarital sex. This may result in ignorance of safe sex practices and a lack of proper public health education by the authorities and parents; this includes knowledge and awareness about sexually transmitted diseases (STDs), modes of transmission, protection methods, and sources of information about STDs.

Methods: This is a cross-sectional study, a Quick Response (QR)-code-based survey. A standard web-based questionnaire was electronically delivered to our enlisted sample. The statistical analysis started by transferring data from the Excel spreadsheet (Microsoft Corporation, Redmond, Washington, United States) to the SPSS software program. We used one-way ANOVA to compare mean scores between the various groups. And we used the Pearson correlation coefficient to assess the association of age with the score. Significance was established at a p-value of 0.05 or less with a 95% confidence interval. All statistical calculations were performed using IBM SPSS Statistics for Windows, Version 27.0 (Released 2020; IBM Corp., Armonk, New York, United States).

Results: The study analysed the sociodemographic characteristics and STD knowledge of 608 participants. Findings revealed a balanced gender distribution, 52.8% male and 47.2% female, the majority being single (72.0%) and with a university education (72.0%). Knowledge gaps were identified, such as confusion between genital herpes and HIV, limited understanding of chlamydia transmission, and misconceptions about human papillomavirus (HPV) and HIV. No significant differences were found based on sex, age, marital status, or father’s education. However, higher education of mother correlated with significantly higher knowledge scores (p < 0.0001).

Conclusion: This study shed light on the limited knowledge and misconceptions surrounding STDs in Taif city. The findings highlighted knowledge gaps, including confusion between different STDs and misconceptions about transmission modes. The results revealed a positive correlation between higher maternal education and increased knowledge scores. These findings underscore the urgency for health authorities to develop awareness campaigns and educational initiatives to promote accurate information and foster healthier attitudes toward sexual activity in these regions.

## Introduction

Sexually transmitted diseases (STDs) refer to a variety of clinical syndromes and infections caused by pathogens that can be acquired and spread through sexual activity [1]. Today, STDs comprise diseases caused by viruses, bacteria, fungi, protozoa, helminths, and arthropods [2]. The key problem with these illnesses is that patients may show no symptoms and go undiagnosed [3]. However, certain sexually transmitted infections (STIs) can harm women's reproductive systems permanently if left untreated, and they can be transmitted from mother to child during pregnancy and childbirth. The World Health Organization estimates that more than a million STDs are acquired every day [4]. 

There is a lack of information regarding both STD epidemiology and the awareness level of prevention in Islamic nations where homosexuality and extramarital sex are forbidden [5]. A study that used a different questionnaire in Saudi Arabia in 2016 showed that participants are unaware of the types, modes of transmission, and methods of STD protection [6]. 

This study uses a previously validated questionnaire, called the STD knowledge questionnaire, and aims to explore knowledge and awareness about STDs among Taif city residents, including modes of transmission, protection methods, and sources of information. More importantly, the study encourages health authorities to establish more awareness campaigns and educational programs about STDs.

## Materials and methods

This is a cross-sectional study that used a Quick Response (QR)-code-based survey. This was carried out over 16 weeks from March 27 to May 17, 2023, and targeted the population of Taif city.

Sampling and population

The sufficient sample size that represents Taif city's population is 385, calculated using an online sample size calculator (Raosoft Inc., Seattle, Washington, United States) with a total population of 1,750,000 and a marginal error equal to 5%, with a confidence level of approximately 95%. We included all individuals who had access to the web, were aged 18 and older, and lived in Taif city, and excluded individuals who did not have access to the web, were younger than 18, or did not live in Taif city. To ensure the eligibility of every individual, we determined the participants’ age and residence. The study was approved by the Scientific Research Ethics Committee at Taif University (approval number: 44-286).

Tools and data collection procedure

The questionnaire used was distributed via multiple data collectors; the data collectors filled the questionnaire electronically, and they were instructed on whom to reach out to and how to properly fill the questions, without influencing their responses. A previously validated web-based questionnaire was delivered to our randomly selected sample by data collectors. The questionnaire we used was the Sexually Transmitted Disease Knowledge Questionnaire (STD-KQ) [7] with 27 questions and with six more demographic questions at the beginning to ensure eligibility and to analyze the data. The first section focuses on social and demographic information, including gender, age, educational level of the respondent, educational level of both parents, and marital status. The second section evaluates the knowledge of STDs. The questionnaire was translated into Arabic and validated in 2018 [3].

Statistical analysis

Simple descriptive statistics of the sociodemographic characteristics and other categorical variables in the form of frequencies and percentages were calculated and tabulated. For continuous variables, means and standard deviations were reported as measures of central tendency and dispersion, respectively.

For the STD-KQ, the total score of the participants was calculated by adding the number of correct responses. A score of 1 was given for each correct response. False is the correct response for these items: 1, 2, 5, 7, 10, 11, 13, 15, 16, 17, 18, 19, 20, 21, 22, 23, 24, 25, 26. True is the correct response for the remaining items: 3, 4, 6, 8, 9, 12, 14, 27. Thus, total scores range from 0-27.

To find the association of the total score with the sociodemographic characteristics, one-way ANOVA was applied and interpreted as the statistical method of choice for comparison of mean scores between the various groups. To assess the association of age with score, the Pearson correlation coefficient was calculated. Significance was established at a p-value of 0.05 or less with 95% confidence interval. All statistical calculations were performed using IBM SPSS Statistics for Windows, Version 27.0 (Released 2020; IBM Corp., Armonk, New York, United States).

## Results

Sociodemographic characteristics

The data provided in Table 1 represents the counts, percentages, mean, and standard deviation for various sociodemographic characteristics including sex, age, marital status, subject's education, father's education, and mother's education. Out of the total 608 participants, 287 (47.2%) were female, while 321 (52.8%) were male. The ages of the participants ranged from 18 years to 41 years with a mean age of 25.8 years and a standard deviation of 6.1 years. Regarding marital status, the majority of participants (72.0%) were single. A significant proportion of participants were married (24.0%), while smaller percentages were divorced (2.6%) or widowers/widows (1.3%).

**Table 1:** Sociodemographic Characteristics of the Participants

Sociodemographic Data		N	%	Mean	Standard Deviation
Sex	Male	321	52.8%		
	Female	287	47.2%		
Age				25.8 years	6.1 years
Marital status	Single	438	72.0%		
	Married	146	24.0%		
	Divorced	16	2.6%		
	Widower/ Widow	8	1.3%		
Subject's education	Illiterate	6	1.0%		
	Primary	10	1.6%		
	Middle school	14	2.3%		
	Secondary	140	23.0%		
	University	438	72.0%		
Father's education	Illiterate	71	11.7%		
	Primary	64	10.5%		
	Middle school	57	9.4%		
	Secondary	159	26.2%		
	University	257	42.3%		
Mother’s education	Illiterate	120	19.7%		
	Primary	69	11.3%		
	Middle school	69	11.3%		
	Secondary	118	19.4%		
	University	232	38.2%		

Participants' educational backgrounds varied across different levels. A small percentage of participants (1.0%) were categorized as illiterate and filled out the questionnaire verbally. Primary education was completed by 10 participants (1.6%), followed by 14 participants (2.3%) with a middle school education. The majority of participants (72.0%) had pursued a university education, indicating a higher level of educational attainment. Information regarding specific fields of study was not provided in the table.

The educational levels of the participants' fathers showed a diverse distribution. The highest proportion of fathers had completed a university education (42.3%), followed by those with secondary education (26.2%). A smaller percentage of fathers were illiterate (11.7%), had a primary education (10.5%), or had a middle school education (9.4%). The educational background of fathers mirrored the diverse educational landscape of the participants. Similar to the fathers, the mothers of the participants exhibited a range of educational backgrounds. The highest percentage of mothers had a university education (38.2%). Additionally, 19.7% of mothers were illiterate, while 11.3% had completed primary school, 11.3% completed middle school, and 19.4% completed secondary school.

The sample displayed a relatively equal distribution between males and females. The majority of participants were single, and a significant proportion had pursued a university education. The educational levels of both fathers and mothers varied, with a considerable number attaining a university education. These findings provide valuable insights into the sociodemographic composition of the participant sample and may be relevant for further analyses and interpretation of study results.

Response to the STD-KQ

Table 2 demonstrates that the majority of participants (55.8%) correctly recognized that having another STD increases the risk of acquiring HIV. This indicates a satisfactory understanding of the interrelationship between different STDs. Only 16.3% of participants correctly recognized that open sores on the genitals do not develop soon after HIV infection. This finding highlights a common misconception regarding the early symptoms of HIV. Only 41.4% of participants correctly identified that genital herpes is not caused by the same virus as HIV. This indicates a lack of awareness about the shared viral cause between these two diseases. Approximately 45.1% of participants correctly identified that human papillomavirus (HPV) is not caused by the same virus as HIV. However, a notably smaller proportion of participants (31.6%) knew that HPV cannot cause HIV, indicating some confusion or misinformation on this topic.

**Table 2:** Response of the Participants to the STD-KQ STD-KQ: Sexually Transmitted Disease Knowledge Questionnaire; STD: sexually transmitted disease; HPV: human papillomavirus

Questions From STD-KQ	Incorrect		Correct Response	
	N	%	N	%
Genital herpes is caused by the same virus as HIV.	356	58.6%	252	41.4%
Frequent urinary infections can cause Chlamydia.	503	82.7%	105	17.3%
There is a cure for gonorrhea.	299	49.2%	309	50.8%
It is easier to get HIV if a person has another STD.	269	44.2%	339	55.8%
Human Papillomavirus (HPV) is caused by the same virus that causes HIV.	334	54.9%	274	45.1%
Having anal sex increases a person’s risk of getting Hepatitis B.	358	58.9%	250	41.1%
Soon after infection with HIV, a person develops open sores on his or her genitals (penis or vagina).	509	83.7%	99	16.3%
There is a cure for Chlamydia.	371	61.0%	237	39.0%
A woman who has genital herpes can pass the infection to her baby during childbirth.	359	59.0%	249	41.0%
A woman can look at her body and tell if she has gonorrhea.	480	78.9%	128	21.1%
The same virus causes all of the STDs.	309	50.8%	299	49.2%
HPV can cause genital warts.	377	62.0%	231	38.0%
Using a natural skin (lambskin) condom can protect a person from getting HIV.	486	79.9%	122	20.1%
HPV can lead to cancer in women.	325	53.5%	283	46.5%
A man must have vaginal sex to get genital warts.	403	66.3%	205	33.7%
STDs can lead to health problems that are usually more serious for men than women	457	75.2%	151	24.8%
A woman can tell that she has Chlamydia if she has a bad smelling odor from her vagina.	487	80.1%	121	19.9%
If a person tests positive for HIV, the test can tell how sick the person will become.	466	76.6%	142	23.4%
There is a vaccine available to prevent a person from getting gonorrhea.	479	78.8%	129	21.2%
A woman can tell by the way her body feels if she has an STD.	500	82.2%	108	17.8%
A person who has genital herpes must have open sores to give the infection to his or her sexual partner.	471	77.5%	137	22.5%
There is a vaccine that prevents a person from getting Chlamydia	497	81.7%	111	18.3%
A man can tell by the way his body feels if he has hepatitis B.	465	76.5%	143	23.5%
If a person had gonorrhea in the past he or she is immune (protected) from getting it again.	474	78.0%	134	22.0%
HPV can cause HIV.	416	68.4%	192	31.6%
A man can protect himself from getting genital warts by washing his genitals after sex.	391	64.3%	217	35.7%
There is a vaccine that can protect a person from getting hepatitis B.	303	49.8%	305	50.2%

A mere 17.3% of participants knew that frequent urinary infections cannot cause chlamydia. This suggests a significant knowledge gap regarding the transmission and causes of this common STD. A relatively low proportion of participants (39.0%) correctly identified that there is no vaccine available for chlamydia. This suggests a need for further education regarding the available treatment options for this prevalent STD. Half of the participants (50.8%) were aware that there is a cure for gonorrhoea. This demonstrates a relatively higher level of knowledge regarding the availability of treatment options for this infection. A significant number of participants (41.1%) acknowledged that engaging in anal sex increases the risk of acquiring hepatitis B. This finding indicates a moderate level of awareness regarding the mode of transmission for this viral infection. Just over 40% of participants (41.0%) correctly understood that a woman with genital herpes can transmit the infection to her baby during childbirth. This finding underscores the importance of disseminating accurate information about the potential risks of transmission during pregnancy. Approximately 38.0% of participants recognized that HPV could cause genital warts. However, a considerably smaller proportion (33.7%) knew that genital warts can be acquired through means other than vaginal sex.

Comparison of knowledge of STDs

Table 3 shows that there was no statistically significant difference in the mean scores between males (Mean = 8.9, SD = 5.7) and females (Mean = 8.4, SD = 5.1), F (1, 606) = 1.587, p = 0.208. The results indicate that the participants' sex did not have a significant impact on their total scores.

**Table 3:** Comparison of Total Score Among Various Sociodemographic Characteristics ^A^One-Way ANOVA; *P<0.05, Significant STD-KQ: Sexually Transmitted Disease Knowledge Questionnaire

Sociodemographic Data		STD-KQ Score		
		Mean	Standard Deviation	P-value^A^
Sex	Male	8.9	5.7	0.208
	Female	8.4	5.1	
Marital status	Single	8.6	5.5	0.942
	Married	8.7	5.2	
	Divorced	9.4	4.6	
	Widower/ Widow	8.9	4.0	
Subject's education	Illiterate	9.8	5.4	0.052
	Primary	11.2	4.9	
	Middle school	6.5	5.4	
	Secondary	7.8	5.6	
	University	8.9	5.3	
Father's education	Illiterate	7.7	5.2	0.169
	Primary	8.1	5.7	
	Middle school	9.3	4.8	
	Secondary	8.4	5.5	
	University	9.1	5.4	
Mother’s education	Illiterate	8.2	5.7	<0.001*
	Primary	8.0	5.2	
	Middle school	8.2	5.0	
	Secondary	7.5	4.9	
	University	9.9	5.4	
Total Score		8.7	5.4	-

The correlation analysis revealed a very weak positive correlation between age and score, with a Pearson correlation coefficient of 0.028. However, this correlation was not statistically significant, as indicated by a p-value of 0.494 (two-tailed test). Thus, the participants' age does not appear to have a substantial impact on their scores.

The analysis revealed no statistically significant difference in the mean scores among participants with different marital statuses: single (Mean = 8.6, SD = 5.5), married (Mean = 8.7, SD = 5.2), divorced (Mean = 9.4, SD = 4.6), and widower/widow (Mean = 8.9, SD = 4.0), F (3, 604) = 0.131, p = 0.942. These findings suggest that marital status did not significantly influence the participants' total scores.

A marginally insignificant difference in the mean scores was observed across different levels of education: illiterate (Mean = 9.8, SD = 5.4), primary (Mean = 11.2, SD = 4.9), middle school (Mean = 6.5, SD = 5.4), secondary (Mean = 7.8, SD = 5.6), and university (Mean = 8.9, SD = 5.3), F (4, 603) = 2.343, p = 0.052. Although not reaching conventional statistical significance (p < 0.05), this finding suggests a potential trend toward higher scores among participants with primary education.

The analysis indicated no statistically significant difference in the mean scores based on the father's education level: illiterate (Mean = 7.7, SD = 5.2), primary (Mean = 8.1, SD = 5.7), middle school (Mean = 9.3, SD = 4.8), secondary (Mean = 8.4, SD = 5.5), and university (Mean = 9.1, SD = 5.4), F (4,603) = 1.616, p = 0.169. Thus, the father's education did not appear to have a significant impact on the participants' total scores.

There was a statistically significant difference in the mean scores among participants with different levels of mother's education: illiterate (Mean = 8.2, SD = 5.7), primary (Mean = 8.0, SD = 5.2), middle school (Mean = 8.2, SD = 5.0), secondary (Mean = 7.5, SD = 4.9), and university (Mean = 9.9, SD = 5.4), F (4, 603) = 5.170, p < 0.001. The results indicate that higher levels of mothers’ education were associated with significantly higher scores.

## Discussion

Our study utilized the STD-KQ to assess participants’ knowledge and understanding of various STDs and related topics. The findings from the questionnaire responses provide valuable insights into the participants’ awareness levels and highlight areas of sufficient knowledge (for example, more than half of the participants acknowledged that it is easier to attract HIV when another STD is already attracted, and that gonorrhoea has a cure), and areas with significant knowledge gaps (for example, the vast majority of participants falsely thought that genital sores soon develop after attracting HIV, and that recurrent urinary tract infection increases the likelihood of attracting chlamydia(.

The study revealed a relatively higher level of knowledge regarding the availability of treatment options for gonorrhoea, with 50.8% of participants correctly recognizing that there is a cure for this infection. The relatively higher knowledge level in this area may be attributed to increased public health efforts to promote awareness about the curability of gonorrhoea.

Furthermore, the finding that a majority of participants (55.8%) correctly recognized that having another STD increases the risk of acquiring HIV demonstrates a satisfactory understanding of the interrelationship between different STDs. This result is consistent with previous studies that have highlighted the association between co-infection with other STDs and increased HIV susceptibility [8,9]. It is encouraging to see this level of awareness, as it underscores the importance of comprehensive sexual health education that addresses the interconnectedness of various STDs.

From the findings of our study, it is also evident that certain misconceptions and knowledge gaps persist among the general population regarding STDs. For instance, only 41.4% of participants correctly identified that genital herpes is not caused by the same virus as HIV. This finding aligns with previous studies that have reported a lack of awareness about the distinct viral causes of these two diseases [10,11]. This knowledge gap underscores the importance of targeted education campaigns to clarify misconceptions and improve understanding regarding the different aetiologies of STDs.

Similarly, the finding that only 17.3% of participants knew that frequent urinary infections cannot cause chlamydia indicates a significant knowledge gap. To address this knowledge gap, educational interventions should focus on providing accurate information about the modes of transmission for chlamydia, emphasizing the importance of safe sexual practices.

Approximately 31.6% of participants mistakenly believed that HPV can cause HIV. This finding aligns with previous studies that have reported confusion or misinformation regarding the relationship between HPV and HIV [12,13]. It is essential to provide accurate information regarding the distinct nature of these infections to dispel misconceptions and ensure that individuals are well-informed about the risks associated with each disease.

Moreover, a relatively low proportion of participants (39.0%) correctly identified that there is no vaccine available for chlamydia. This result indicates a need for further education regarding the available treatment options for chlamydia [14]. While vaccines are available for certain STDs, such as HPV, the absence of a vaccine for chlamydia emphasizes the importance of other preventive measures, including consistent condom use and regular testing.

A significant majority of participants (58.9%) incorrectly believed that having anal sex increases the risk of acquiring hepatitis B. This misconception indicates a lack of awareness about the specific modes of transmission for hepatitis B, which include exposure to infected blood, unprotected sexual contact, and sharing contaminated needles [15]. It is thus essential to educate the public about the transmission routes of hepatitis B, including the risks associated with various sexual practices, to promote safer behaviours and prevent the spread of the disease.

The findings also revealed misconceptions regarding the transmission of STDs. For example, only 41.0% of participants correctly understood that a woman with genital herpes can transmit the infection to her baby during childbirth. This finding aligns with prior studies that have indicated a lack of awareness about the potential risks of perinatal transmission of genital herpes [16]. Comprehensive sexual health education should include information about the modes of transmission for STDs during pregnancy and childbirth to ensure individuals are well-informed about the potential risks and take appropriate preventive measures.

Furthermore, approximately 33.7% of participants incorrectly believed that genital warts can only be acquired through vaginal sex. This misconception highlights a knowledge gap regarding the modes of transmission for genital warts. It is crucial to educate individuals about the various ways in which genital warts can be transmitted, including through oral and anal sex, to ensure a comprehensive understanding of the risks associated with this infection.

STDs are common, but many people do not have enough information about them. This can lead to risky sexual behaviour and the spread of STDs. There are a number of reasons why people may not know much about STDs. Some people may not have received adequate sexual health education, while others may have been taught inaccurate or outdated information. Additionally, some people may be reluctant to talk about STDs because they are seen as taboo or embarrassing.

To address these knowledge gaps, health education programs should focus on providing accurate and up-to-date information about STDs. Incorporating evidence-based interventions, such as interactive workshops, educational campaigns, and comprehensive sexual health curricula can effectively improve knowledge and promote healthy behaviours [17]. Additionally, utilizing various channels for information dissemination, such as social media platforms, websites, and healthcare providers, can reach a wider audience and ensure accessibility to accurate information. Also, encouraging parents to participate in the health promotion process by addressing these issues with their offspring more liberally, thus breaking the cycle as early as when these young people start their sexual lives.

Our study also compared the knowledge of STDs among participants based on various sociodemographic characteristics. Firstly, the analysis of sex differences indicated that there was no statistically significant disparity in the mean scores between males and females. This finding aligns with previous studies such as Burrell et al. [18] and Adeyemi [19], which also reported no significant differences in STD knowledge between genders. Therefore, our study supports the notion that gender does not play a significant role in influencing individuals’ knowledge of STDs.

Regarding age, the correlation analysis revealed a very weak positive correlation between age and the scores. However, this correlation was not statistically significant, indicating that age does not have a substantial impact on participants’ knowledge of STDs. These results are consistent with the study conducted by Smith et al., which found no significant association between age and STD knowledge among their participants [20].

The examination of marital status revealed no significant differences in the mean scores among individuals with different marital statuses. This finding is consistent with the study by El-Tholoth et al., which also reported no significant variations in STD knowledge based on marital status [6]. Thus, marital status does not seem to significantly influence individuals’ knowledge of STDs.

In terms of the subject’s education level, although the difference in mean scores was marginally insignificant, there appeared to be a potential trend toward higher scores among participants with primary education. This finding is consistent with the study by Mwambete et al., which found that individuals with higher levels of education generally exhibited greater knowledge of STDs [21]. However, further research with a larger sample size is warranted to explore this potential trend in more detail.

Regarding the influence of parents’ education, the analysis showed that father’s education level did not have a significant impact on the participants’ total scores. These findings align with the study conducted by Wanje et al., which also found no significant association between father’s education and STD knowledge [22]. However, interestingly, mother’s education level was significantly associated with participants’ knowledge of STDs. Higher levels of mother’s education were associated with significantly higher scores. This finding is consistent with previous research by Koray et al. and supports the notion that maternal education plays a crucial role in shaping individuals’ awareness and understanding of STDs [23].

Limitations

It Is important to note that this study has certain limitations. Firstly, the participants’ responses were based on self-reported knowledge, which may be subject to recall bias or social desirability bias. Additionally, the study sample may not be representative of the general population, as it consisted of a specific group of individuals who were more reachable by the data collectors. Future research should aim to include a more diverse sample to ensure broader generalizability of the findings.

The age range of the participants was between 18 and 41 years, with a mean age of 25.8 years and a standard deviation of 6.1 years. This is considered a limitation for the study because the older age group is underrepresented in comparison to the younger. In terms of marital status, the majority of participants (72.0%) were single, while a significant proportion (24.0%) were married. A lower percentage of participants were divorced (2.6%) or widowers/widows (1.3%). Thus, if the study was conducted on married people only, a different outcome would be expected. Another limitation is that most participants (72.0%) had pursued a university education, indicating a higher level of educational attainment within the sample. Hence, there is a skew towards higher-educated people.

The educational backgrounds of the participants’ fathers and mothers also provide insights into the sociodemographic composition of the sample. The fathers exhibited a diverse distribution of educational levels, with the highest proportion having completed university education (42.3%). Similarly, the educational background of the mothers varied, with the highest percentage having a university education (38.2%). These findings suggest that the participants came from families with diverse educational backgrounds, which may influence their own educational attainment and potentially impact the study outcomes.

## Conclusions

This study shed light on the limited knowledge and misconceptions surrounding STDs in Taif city. The findings highlighted knowledge gaps, including confusion between different STDs and misconceptions about transmission modes. The results revealed a positive correlation between higher maternal education and increased knowledge scores. These findings underscore the urgency for health authorities to develop awareness campaigns and educational initiatives to promote accurate information and foster healthier attitudes toward sexual activity in these regions.
